# Patterns of genomic and allochronic strain divergence in the fall armyworm, *Spodoptera frugiperda* (J.E. Smith)

**DOI:** 10.1002/ece3.8706

**Published:** 2022-03-21

**Authors:** Ashley E. Tessnow, Tyler J. Raszick, Patrick Porter, Gregory A. Sword

**Affiliations:** ^1^ Department of Entomology Texas A&M University College Station Texas USA; ^2^ 14736 Texas A&M AgriLife Research & Extension Center Lubbock Texas USA

**Keywords:** allochronic divergence, fall armyworm, hybridization, sympatric strains

## Abstract

Speciation is the process through which reproductive isolation develops between distinct populations. Because this process takes time, speciation studies often necessarily examine populations within a species that are at various stages of divergence. The fall armyworm, *Spodoptera frugiperda* (J.E. Smith), is comprised of two strains (R = Rice & C = Corn) that serve as a novel system to explore population divergence in sympatry. Here, we use ddRADSeq data to show that fall armyworm strains in the field are largely genetically distinct, but some interstrain hybridization occurs. Although we detected F1 hybrids of both R‐ and C‐strain maternal origin, only hybrids with R‐strain mtDNA were found to contribute to subsequent generations, possibly indicating a unidirectional barrier to gene flow. Although these strains have been previously defined as “host plant‐associated,” we recovered an equal proportion of R‐ and C‐strain moths in fields dominated by C‐strain host plants. As an alternative to host‐associated divergence, we tested the hypothesis that differences in nightly activity patterns could account for reproductive isolation by genotyping temporally collected moths. Our data indicates that strains exhibit a significant shift in the timing of their nightly activities in the field. This divergence in phenology creates a prezygotic reproductive barrier that likely maintains the genetic isolation between strains. Thus, we conclude that it may be ecologically inaccurate to refer to the C‐ and R‐ strain as “host‐associated” and they should more appropriately be considered “allochronic strains.”

## Graphical abstract

The fall armyworm is comprised of two strains (R = Rice & C = Corn) that serve as a novel system to explore population divergence in sympatry. Here, we show that these strains remain genetically distinct in the field, despite occasional hybridization. Our data also indicates that in the field these strains exhibit a significant shift in the timing of their nightly activities, a factor that likely maintains their genetic isolation.

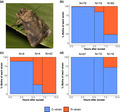

## 1 INTRODUCTION

Understanding speciation and the origin of biodiversity is fundamental to the field of evolutionary biology. Most definitions of speciation require the development of reproductive isolation between populations as a result of disruptive or divergent selection (Mayr, 1942; Seehausen et al., 2014). The most widely accepted scenario for disruptive selection is when two populations become spatially separated, and thus experience different selection pressures as a result of their geographic isolation (Coyne, 1992). However, both disruptive selection and population‐specific directional selection can also act on sympatric populations resulting in divergence. For example, the availability of a novel host plant (Feder et al., 1988; Filchak et al., 2000; Rice, 1984), sexual selection resulting in assortative mating (Turner & Burrows, 1995), and divergent selection acting on phenology (Fukami et al., 2003; Santos et al., 2007, 2011) are all mechanisms that have been shown to lead to population divergence in sympatry. Because speciation takes many generations and is nearly impossible to study in real‐time for multicellular organisms, researchers rely on populations that are undergoing various stages of speciation to gain insights into this process.

The fall armyworm, *Spodoptera frugiperda* (J.E. Smith) (Lepidoptera: Noctuidae), is a moth species native to the Western Hemisphere. In the United States, this insect only overwinters in south Texas and south Florida, and these locations serve as the source for fall armyworms reinvading the northern US and Canada each year (Sparks, 1979). No evidence of a return migration has been found for this species. Thus, at the end of the season, individuals occurring north of these overwintering sites die and their genetic variation is lost (Nagoshi & Meagher, 2008). Importantly, this species is comprised of two morphologically identical but genetically distinct sympatric strains that have been previously described as “host‐associated”. These strains were originally discovered by Pashley (1986) and named for the crops on which they were found, corn and rice (Pashley, 1986). Although this species is highly polyphagous feeding on up to 353 host plants from 76 plant families (Montezano et al., 2018), the larvae of the corn‐strain, or C‐strain, are considered to be primarily associated with corn and sorghum, whereas the rice‐strain, or R‐strain, are more commonly associated with pasture grasses, turf grass, and rice.

Although the two fall armyworm strains can be consistently identified using genetic markers, hybridization has been reported in both the lab and field, with field studies suggesting that 16% of moths collected show inconsistencies between multiple strain‐specific genetic markers (Prowell et al., 2004). In these field assessments, the majority of putative hybrids have the maternally inherited mitochondrial markers from the R‐strain, indicating a directional mating bias (Nagoshi, 2010; Nagoshi, Meagher, Nuessly, et al., 2006; Prowell et al., 2004). In the lab, single pair matings have been conducted in both directions with evidence of reduced fertility among interstrain hybrids (Dumas et al., 2015; Kost et al., 2016). Still, these genetic markers have not become homogenous across the two strains, suggesting these strains remain genetically distinct despite occasional gene flow.

Despite being described as “host‐associated”, the host ranges of the two fall armyworm strains largely overlap, with evidence of asymmetric host use between strains (Groot et al., 2010, 2016). So, although the C‐strain is more commonly found in association with corn and sorghum, the R‐strain can also be found feeding on these hosts. It is uncommon, however, for the C‐strain to be found feeding on smaller grasses such as pasture grasses and turf (Machado et al., 2008; Nagoshi, 2010). Several studies have used behavioral assays to assess whether the strains show a strong preference or fitness benefit when fed on different host plants, but no consistent differences have been found (Meagher et al., 2004; Pashley, 1988b; Pashley et al., 1995). This frequent overlap in habitat use and lack of strong host association suggests that differences in host plant use are unlikely to be the only factor maintaining genetic differentiation between these strains. Given the limited empirical evidence that host plant differences are involved in divergence, several studies have suggested that it may be more appropriate to refer to these strains as incipient species or genetic forms rather than host strains (Dumas et al., 2015; Juárez et al., 2014; Kergoat et al., 2012).

As an alternative to host‐association, allochrony in nightly mating has been hypothesized as a mechanism underlying strain divergence. Pashley et al. (1992) found that strains exhibited differences in the timing of their mating activities, with the C‐ strain becoming active early in the scotophase (i.e., night) and the R‐strain becoming active much later in the scotophase (Pashley et al., 1992). This temporal difference has been observed in subsequent laboratory mating assays (Hänniger et al., 2017; Schöfl et al., 2009), and linked to heritable polymorphisms in the circadian rhythm modulator gene, *vrille* (Hänniger et al., 2017). If this temporal divergence also occurs in the field, the fall armyworm could be an excellent study system for assessing incipient allochronic divergence and speciation in action.

In this study, we used double digest restriction site‐associated DNA sequencing data to better elucidate the patterns of divergence and gene flow between *S*. *frugiperda* strains collected from five locations across the central US. We then used temporal collection data to test the hypothesis that C‐ and R‐ strain fall armyworm moths in the field exhibit significant temporal differences in their nightly activity periods. Our study provides new insights into the patterns of genomic divergence and reproductive isolation of fall armyworm strains in the United States.

## 2 METHODS

### 2.1 Central US insect collection for sequencing

*Spodoptera frugiperda* moths were collected using universal moth traps baited with Scentry PSU 2‐component lures (Scentry Biologicals) and containing Hercon Vaportape. Strains do not significantly differ in their attraction to pheromone lures, and therefore no bias in strain sampling is expected (Unbehend et al., 2014). Each trap was placed in or around corn and sorghum fields at five locations across the central US. Multiple sampling times were selected throughout the year that roughly corresponded to seasons when moths were present at each location (Table 1). During each sampling period, traps were checked daily until a minimum of 24 moths were captured. At sites in the Lower Rio Grande Valley, larvae were occasionally collected by hand from nearby host plants. All sampled insects were immediately preserved in 95% ethanol and stored at 4°C until shipment to Texas A&M University in College Station, TX. Upon arrival, all specimens were stored at −80°C until DNA extraction. In total, 426 moths were sequenced across the 2 years.

**TABLE 1:** Collection location and date for all sequenced fall armyworm samples. The most common crop type surrounding each trap is listed as host plant. The numbers of individuals from each collection is given both as the number per predetermined strain mitochondrial haplotype (R‐ or C‐) and the number of total individuals from each collection (R‐ + C‐)

Location	GPS coordinates (Lat, Long)	Date	Host plant	# Sequenced		
				R‐	C‐	Total
Lower Rio Grande Valley, TX	26.1556, −97.9618 & 26.2099, −97.5432	March 13–15, 2017	Sorghum	6	16	22
	26.1556, −97.9618	November 16, 2017	Sorghum	12	0	12
	26.0924, −97.8814 & 26.0869, −98.2601	May 10–11, 2018	Sorghum	7	15	22
	26.1556, −97.9618	July 12–13, 2018	Sorghum	22	1	23
	26.1556, −97.9618	December 11–12, 2018	Sorghum	16	2	18
Corpus Christi, TX	27.7827, −97.5621	April 18–20, 2017	Sorghum	20	1	21
	27.7827, −97.5621	September 28–30, 2017	Sorghum	16	3	19
	27.7827, −97.5621	May 12–13, 2018	Sorghum	8	10	18
	27.7827, −97.5621	July 10–11, 2018	Sorghum	2	12	14
	27.7827, −97.5621	October 7–8, 2018	Sorghum	1	12	13
College Station, TX	30.6206, −96.3617	May 25–26, 2017	Sorghum	13	10	23
	30.6206, −96.3617	July 6–7, 2017	Sorghum	0	16	16
	30.6206, −96.3617	October 23–27, 2017	Sorghum	12	0	12
	30.6206, −96.3617	May 16–18, 2018	Sorghum	11	7	18
	30.6206, −96.3617	June 28–29, 2018	Sorghum	5	10	15
	30.6206, −96.3617	October 19–24, 2018	Corn	12	0	12
Lubbock, TX	33.6912, −101.8259	May 24–31, 2017	Corn	0	15	15
	33.6912, −101.8259	June 21–27, 2017	Corn	7	16	23
	33.6912, −101.8259	September 21, 2017	Corn	9	13	22
	33.6912, −101.8259	May 2, 2018	Corn	0	12	12
	33.6912, −101.8259	June 12, 2018	Corn	1	11	12
	33.6912, −101.8259	September 13, 2018	Corn	5	13	18
Rosemount, MN	44.7069, −93.1068	September 12–14, 2017	Corn	8	18	26
	44.7069, −93.1068	August 21. 2018	Corn	0	20	20

### 2.2 DNA extraction

Prior to DNA extraction, the thorax was isolated from each specimen and surface sterilized in 95% ethanol. Tissues were tapped dry, placed individually in 2‐ml Eppendorf tubes, and then frozen in liquid nitrogen. Sterilized plastic pestles were used to macerate the frozen thorax tissue. The Qiagen Gentra Puregene Tissue Kit was used to extract DNA following the manufacturer's protocol. The concentration of each DNA sample was measured on a NanoDrop spectrophotometer and all samples were diluted to a concentration of 50 ng/μl.

### 2.3 Initial strain haplotype determination

After DNA extraction, strains were assigned using two known RFLPs in the cytochrome c oxidase subunit I (COI) mitochondrial gene (Levy et al., 2002; Nagoshi, Meagher, Adamczyk, et al., 2006). Briefly, the primer pair JM‐76/JM‐77 was used to amplify a 568 bp fragment of COI (Levy et al., 2002). Then 4 μl of the PCR product was added to both 2.5 μl of SacI (New England BioLabs) and 2.5 μl of MspI (New England BioLabs) diluted to their optimal working concentrations. Reactions were incubated at 37°C for 1 h, and the products were run on a 1.8% agarose gel. The amplified C‐strain mtDNA is cut once by MspI, and not by SacI while the R‐strain mtDNA shows the reciprocal pattern (Nagoshi, Meagher, Adamczyk, et al., 2006). Based on the cut patterns of both restriction enzymes, each individual was assigned as having either a C‐strain or an R‐strain mitochondrial haplotype. After haplotype determination, DNA was stored at −20°C until sequencing.

### 2.4 DNA sequencing, SNP calling, and filtering

DNA samples were sent to Texas A&M AgriLife Genomics and Bioinformatics Services (TxGen) for quality control, library preparation, and double digest restriction‐site‐associated DNA sequencing (ddRADseq) (Peterson et al., 2012). Prior to library prep, DNA was purified using the Agencourt AMPure XP purification system. Libraries were prepared by digesting the total genomic DNA with MseI and EcoRI restriction enzymes, and 300 to 500 bp fragments were size selected for sequencing. Each fragment was ligated to standard Illumina adapters, sequencing primers, and multiplexing indexes. All sequencing was conducted on the Illumina NovaSeq 6000 to yield 150 bp paired end reads. Sequence cluster identification, quality prefiltering, base calling, and uncertainty assessment were then conducted using Illumina's NCS 1.0.2 and RFV1.0.2 software with default parameter settings.

TxGen provided the demultiplexed raw sequences and FastQC v.0.11.7 reports. FastQC reports were reviewed to ensure suitable quality, and then sequences were uploaded into the Texas A&M High Performance Research Computing “Ada” cluster for bioinformatic analyses. All sequences are now available through the NCBI Sequence Read Archive (See Data Accessibility Statement).

On average, 1.83 million 150 bp reads were obtained in each individual ddRAD library. This translated to an average of 275MB of sequence data per sample before filtering.

FastQ Screen v.0.14.0 with the BWA aligner was used to align raw reads to both the C‐strain and R‐strain published *S*. *frugiperda* genomes (Gouin et al., 2017). Sequences that did not match uniquely to one or both genomes were removed to clear the remaining sequences of all potential contaminant DNA (e.g., bacteria, pathogens). Forward and reverse reads were then matched together using the repair function in BBMAP v.3.8.08 (Chaisson & Tesler, 2012). After filtering out contaminant DNA and DNA that matched multiple locations in the *S*. *frugiperda* genome, an average of 42.5% of the initial raw reads were retained for SNP calling.

Genomic loci that contained SNPs were identified using the dDocent v.2.2.16 SNP‐calling pipeline (Puritz et al., 2014). In brief, dDocent removed low quality bases using Trimmomatic, and then mapped reads to the Liu et al. (2019) published chromosome map for *S*. *frugiperda* using BWA. The program FreeBayes then identified genomic loci containing SNPs and indels, and these variants were concatenated into a single VCF file. Our initial VCF file contained 441,437 variants.

Variants were filtered using VCFtools v.0.1.16 (Danecek et al., 2011). Specifically, all indels were removed and the remaining SNPs were filtered for a minimum PHRED score of 30. Only SNPs that were present in all individuals at a minimum of 3x coverage were kept in the final dataset. Finally, the dDocent_filters script (https://github.com/jpuritz/dDocent/blob/master/scripts/dDocent_filters) was run to complete SNP filtering. After filtering, the VCF file was manually examined and 236 SNPs did not map to a specific chromosome but rather to an ‘unplaced_scaffold.’ These unmapped SNPs were removed, leaving 5439 mapped SNPs in the final dataset.

### 2.5 Analysis of molecular variance

The VCF file was uploaded into RStudio v.3.6.1 using R/vcfR v 1.9.0 (Knaus & Grünwald, 2017). To determine which collection factors (sampling year, location, season, and strain) significantly impacted the population structure of *S*. *frugiperda*, we carried out an analysis of molecular variance (AMOVA) using R/poppr v.2.8.3 (Kamvar et al., 2014). We used a Monte Carlo test with 1000 random permutations to determine the statistical significance of each factor in the AMOVA.

### 2.6 Population structure and strain admixture analyses

To examine the population structure within fall armyworm samples, the VCF file was converted to a biallelic.bed (Plink Binary Biallelic Genotype Table) file and then to Eigenstrat format using PLINK v.1.07 (Purcell et al., 2007) and EIGENSOFT v.7.2.1 (Price et al., 2006), respectively. We then used the *smartpca* function within EIGENSOFT 7.2.1 to conduct a smart principal component analysis that identified and removed outliers in the dataset caused by population stratification (Patterson et al., 2006; Price et al., 2006). This program calculated Tracy‐Widom statistics to determine the number of significant eigenvalues, or principal components, within the PCA. The PCA results were then plotted using R/ggplot2 (Wickham, 2016). Because the smartPCA revealed two distinct SNP based population clusters that corresponded to host strains, putative hybrids were identified as individuals that either did not fit neatly into the R‐strain or C‐strain SNP clusters, or individuals that had a mismatch between their mtDNA and SNP‐based strain assignments.

To determine if the putative hybrids showed significant evidence of interstrain admixture, outgroup *f3* statistics were calculated using the 3‐populations test function (qp3Pop) in AdmixTools v.5.0 (Patterson et al., 2012). In this test, pure‐strain individuals from the R‐strain and the C‐strain were defined as the ancestral populations, and each putative hybrid individual was uniquely assessed for admixture using the model *f3*(C‐strain, R‐strain; putative hybrid individual). Only individuals that had significantly negative *f3* values were considered true hybrids.

The program ADMIXTURE 1.3.0 was run using default parameters to determine the probability that individual moths were assigned to one or more genetically distinct groupings (Alexander & Lange, 2011). The *K*‐values input ranged from 1 to 15, and the optimal value of *K* was determined as the run that resulted in the lowest cross validation (CV) error. The browser‐based program CLUMPAK was used to visualize the population assignment of all individuals (Kopelman et al., 2015).

### 2.7 Assessing genomic patterns of strain divergence

Using the smartPCA and admixture results, each individual was assigned to the R‐strain or the C‐strain based on their SNP groupings. Individuals with significant evidence of admixture from the *f3* test were removed from this analysis. To determine the level of divergence between strains at each SNP locus, a fixation index, or *F*_ST_ value, was calculated for every mapped SNP using R v.3.6.2/genepop (Rousset, 2008). A Manhattan plot visualizing the chromosome position of each SNP and its associated *F*_ST_ values was created using R v.3.6.2/qqman (Turner, 2018).

SNPs with *F*_ST_ values >0.7, or that appeared as outliers on the Manhattan plot, were identified and mapped back to the published chromosome map (Liu et al., 2019) using Geneious v.11.0.2. After mapping, a 501 bp DNA fragment that included the SNP and 250 bps up and downstream of the variant was extracted from the genome. In several cases, divergent SNPs were in close proximity to one another and were grouped together. In these case, a DNA segment 250 bp upstream of the most 5′ SNP and downstream of the most 3′ SNP was extracted. Each DNA sequence was then uploaded and searched in the NCBI insect nucleotide database (taxid:6960) using megablast to identify any similar, previously characterized, nucleotide sequences.

### 2.8 Assessing temporal differences in strain activity in the field

Universal bucket traps (Great Lakes IPM) baited with 2‐component fall armyworm lures (Scentry) were placed more than 100 m apart in agricultural fields at two Texas A&M University AgriLife research facilities; Lubbock, TX (GPS: 33.6944, −101.8249) on September 2–4, 2020, and College Station, TX (GPS 33.6944, −101.8249) on 17 June 2021. At the time of trapping the light cycles in College Station and Lubbock were 15L:9D and 14L:10D, respectively. Moths were removed from the traps and preserved in 95% ethanol after three time intervals; 5 h after sunset, 7 h after sunset, and again after sunrise. These intervals are referred to as the early, middle, and late time periods. In Lubbock, moths were collected from six traps across three different nights, and in College Station, moths were collected from 10 traps across one night. Nearby host plants were recorded for every trap (Table A1). Once back in the lab, all moth samples were stored in 95% ethanol at −20°C until DNA extraction.

Moths were visually inspected to verify their identity as fall armyworms. The thorax was then isolated using a sterile razor, placed in a 2‐ml tube, and DNA was extracted as described above. DNA was quantified using either a fluorometer (DeNovix) or a Multiskan Go spectrophotometer (ThermoFisher) and diluted to a concentration of 20 ng/µl.

TaqMan assays were used to determine the allele present at three previously described diagnostic SNP loci for each strain referred to as SNP A, B, and C (Tessnow et al., 2021). Briefly, 1 µl of diluted DNA was added to 5 µl of TaqMan Genotyping Master Mix (ThermoFisher), 3.5 µl of nuclease free water, and 0.5 µl of a Custom TaqMan SNP genotyping assay (ThermoFisher). Reactions were held at 95°C for 10 min then cycled 40 times between 95°C for 15 s and 60°C for 1 min. Fluorescence was recorded after each cycle. qPCR for samples collected in Lubbock and College Station was conducted on a CFX384 (BioRad) and QuantStudio 3 (ThermoFisher) Real‐Time PCR detection system, respectively. Cq values for real time PCR were calculated using either CFX Maestro v. 4.1.2434.0124 or the QuantStudio Design & Analysis Software v. 1.5.2. Data were then exported into Excel v.16.53 to determine the strain based on the delta Cq between the two flourophores (Tessnow et al., 2021).

After strain determination, generalized linear models with a binomial distribution and logit link were run to determine the effects of collection time, trap position, and night (Lubbock only) on the probability of collecting C‐ or R‐ strain individuals at each of the two locations. In both cases, the only factor that was significant was the collection time, so the data from both locations were combined and anther generalized linear model was used to assess the effects of time, location, and the time × location on the probability of collecting each strain. All statistical analyses were conducted in JMP Pro v.15.0 (SAS Institute Inc.).

## 3 RESULTS

### 3.1 Factors that contribute to genetic variation in sequencing data

**FIGURE 1:**
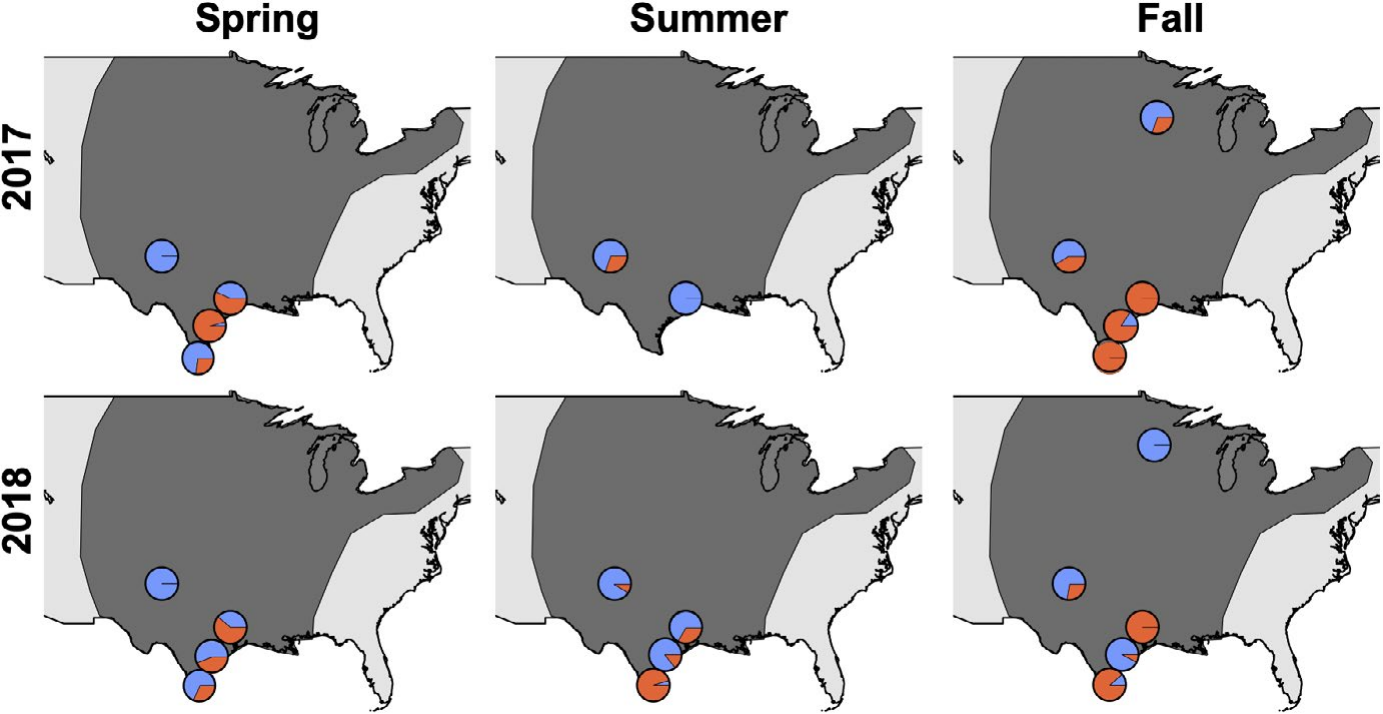
Proportion of individuals with C‐strain (blue) or R‐strain (orange) mtDNA in each moth collection. The dark grey overlay illustrates the proposed central US flyway for fall armyworm moths (inferred from Westbrook et al. (2016))

Prior to sequencing, we determined the relative proportion of each central US collection that was comprised of the R‐strain and C‐strain individuals using mtDNA haplotypes. Although traps were placed in and around corn and sorghum fields which are typically considered C‐strain host plants (Pashley, 1988a, 1988b), we found that most locations contained a mix of both C‐ and R‐strain haplotypes. Unexpectedly, several collections, especially during the fall season, were solely comprised of individuals with R‐strain haplotypes (Figure 1). Because we had collected a representative sample of both host strains during most collection times, a mix of individuals comprising both host strains were sequenced (Table 1).

Although there were several potential sources of genetic structure in our dataset (year, location, season, and strain), our AMOVA revealed predetermined host strain haplotype was the only factor that significantly accounted for genetic variability in the data (Table 2, *ϕ* = 0.09, *p* < .001). The variables year, location, and season were not significant (Table 2).

**TABLE 2:** Sources of genetic variation between all fall armyworm samples determined by an analysis of molecular variance (AMOVA). The factors assessed include collection year, sampling location, sampling season, and host strain. Host strain was the only collection factor that contributed significantly to the population structure of *S*. *frugiperda*

Source of variance	df	% Variance	*ϕ*‐Statistic	*p*‐Value
Between years	1	−0.44	−0.004	.618
Within year between Locations	8	0.98	0.01	.209
Within locations between seasons	14	−2.06	−0.021	.788
Within season between strains	16	9.17	0.09	<.001*****
Between samples	386	5.97	0.065	<.001***
Within samples	426	86.40	0.136	<.001***

Note

^***^ indicates *p*‐value is ≤.001

**FIGURE 2:**
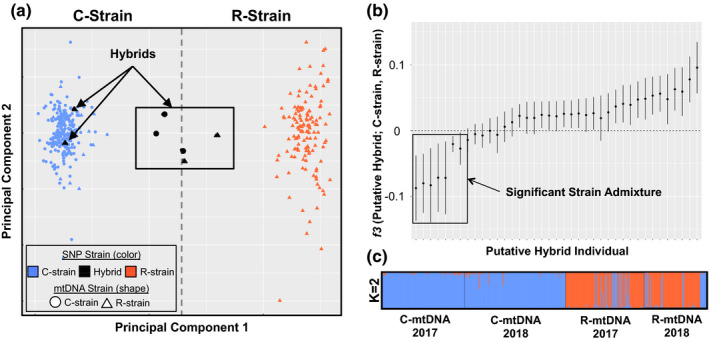
SNP data shows genetic differentiation between strains, however, some admixture is evident. (a) smartPCA results split C‐strain (blue) and R‐strain (orange) individuals into two clusters along principal component 1. Five individuals fall in between the two clusters, and 34 individuals have the R‐strain mtDNA but are grouped in the C‐strain SNP cluster. Individuals that are marked in black exhibit significant strain admixture (*f3* < 0). (b) Outgroup *f3* statistics plotted for each putative hybrid individual. Individuals with an *f3* < 0 are significantly admixed. (c) ADMIXTURE plot (*K* = 2) for all individuals split according to their mtDNA and collection year. Each bar illustrates the probability of assignment to one of two genetically distinct groups for a single individual

The major effect of host strain haplotype on genetic structure was further supported by a smartPCA conducted on the SNP dataset. In this analysis, the data clustered into two distinct groupings along PC1 that roughly corresponded with the predetermined mitochondrial haplotypes (Figure 2a). Other principal components did not identify any additional population groupings. When conducting the SmartPCA, 33 samples were removed as outliers due to cryptic relationships (genetically too similar within collections). Roughly half of these outliers were from the spring collections conducted in the Lower Rio Grande Valley, in which caterpillars were collected as opposed to moths. Since, it is reasonable that some sibs or half sibs were collected when sampling caterpillars from the same fields, we continued our analysis with only the remaining 393 unrelated individuals.

In addition to conducting a PCA, Tracy‐Widom statistics were calculated to evaluate the statistical significance of each eigenvalue or principal component (Patterson et al., 2006). We found that only the first principal component was statistically significant (TW = 405.962, *p* < .0001), while all other principal components had *p*‐values greater than .05. Together, this indicates that the only factor contributing to genetic diversity in our data was strain. Finally, as part of the smartPCA, an ANOVA was run using each of the first 10 eigenvectors to determine if the population assignments significantly explained the genetic differentiation across the first 10 principal components. Here, we assigned individuals to one of two populations (R‐strain or C‐strain) based on their mtDNA haplotypes. The ANOVA stats were then summed across eigenvectors, giving a chi‐square distribution with the df = 10. Significant genetic differentiation in the dataset could be explained by strain assignment (χ^2^ = 471.194, *p* < .0001).

Because multiple lines of evidence indicated that *S*. *frugiperda* population structure across the central US was explained entirely by strain, further analyses focused on admixture and genetic differentiation between strains.

### 3.2 Admixture between host strains

Although the two clusters in the PCA could largely be explained by strain mtDNA haplotypes, there were 34 individuals that showed a mismatch between the mtDNA haplotypes and their SNP cluster assignment. One hundred percent of these mismatched individuals contained R‐strain mtDNA, but clustered within the C‐strain SNP cluster. Additionally, two individuals with R‐strain mtDNA and three individuals with C‐strain mtDNA mapped directly in between the two SNP clusters on the smartPCA (Figure 2a). This assemblage of 39 individuals comprised of mismatches and those that did not group were classified as putative hybrids.

*f3* statistics were calculated to determine if these putative hybrids exhibited significant admixture between the two strain source populations. In this analysis, *f3* values significantly lower than zero indicate significant admixture between two source populations. We found that all five individuals that did not neatly group with either SNP cluster exhibited significant admixture between the two host strains. Additionally, two individuals that grouped with the C‐strain SNP cluster but carried the R‐strain mtDNA (mismatches), exhibited significant strain admixture (Figure 2b). These seven individuals will henceforth be referred to as the hybrids and are indicated as such on Figure 2a.

ADMIXTURE analysis was then conducted to calculate the probability of individuals assigned to one or more (*K*) genotypic groups. The lowest cross‐validation error (CV) occurred when *K* = 2 or with two genotypic groups. *K* = 3 or higher did not show any additional population resolution. These two genotypes largely corresponded with the predetermined strain haplotypes (Figure 2c). Slightly more admixture was detected among individuals with the R‐strain mtDNA compared to those with C‐strain mtDNA.

### 3.3 Genomic patterns of strain divergence

**FIGURE 3:**
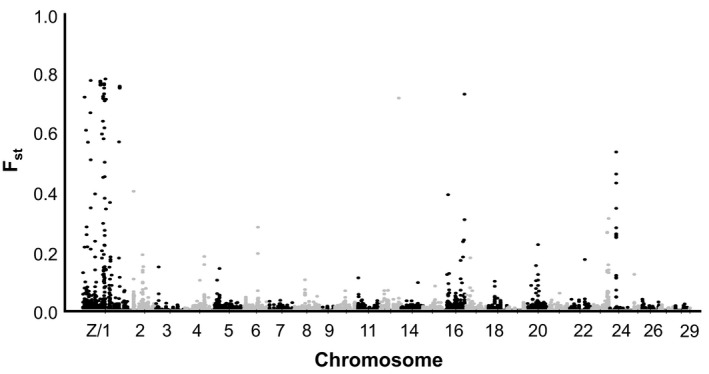
Manhattan plot illustrating locus specific *F*_ST_ values differentiating fall armyworm strains for every SNP identified. Here, fall armyworm strains were defined as individuals that grouped in either the C‐strain or R‐strain SNP cluster as seen in Figure 2a

To determine the level of divergence between strains at every SNP locus, we calculated the fixation index, or *F*_ST_ values, for each of the 5439 high‐quality mapped SNPs. We then visualized patterns of divergence across the genome by plotting the *F*_ST_ for every SNP across chromosomes using a Manhattan plot (Figure 3). The Manhattan plot indicated that the majority of divergent SNPs, those with *F*_ST_ values closest to 1, were located on the Z‐chromosome, but some divergence may also be seen on chromosomes 12, 16, and 24 of the Liu et al. (2019) chromosome map. Of the SNPs with *F*_ST_ values >0.7, 28 of 30, or 93% were located on the Z‐chromosome (Table A2).

We then calculated global *F*_ST_ values across all 5439 SNPs in the genome and found that there was moderate genetic differentiation between the C‐ and R‐strains (*F*_ST_ = 0.108). We further characterized SNPs with *F*_ST_ values >0.7. This cutoff was chosen arbitrarily but indicates high strain identity when considering that putative hybrids are evident in the dataset. We found that most of the highly divergent SNPs that we identified that might map to coding regions of the genome would result in synonymous mutations. However, one group of SNPs may disrupt the function of a suppressor of cytokine signaling gene located on the Z‐chromosome (Table A2). Further work is required to explore the functional significance of these findings. Since, ddRADseq is designed to randomly identify neutral SNP markers across the genome, SNPs with high *F*_ST_ values may be linked to genomic regions that show high levels of strain divergence, even if these SNPs do not cause changes to the protein coding sequence.

### 3.4 Temporal differences in strain activity

**FIGURE 4:**
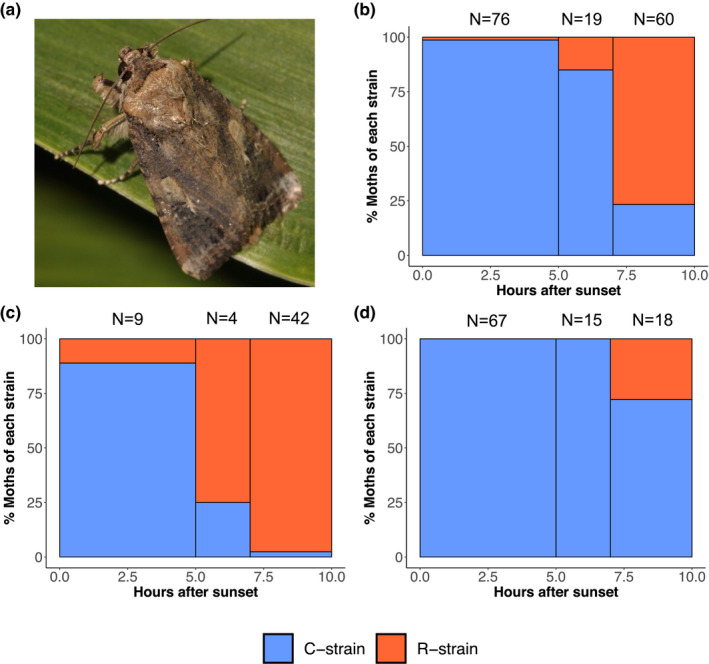
Percent of moths captured comprising each strain during three night time intervals, 0–5 h after sunset, 5–7 h after sunset, and 7 h after sunset until sunrise. These captures are split into three groups: (b) all moths collected and pooled across both locations, (c) just moths collected in College Station, and (d) just moths collected in Lubbock. The total number of moths collected at each time point is indicated above each bar. (a) Photo of a male fall armyworm moth resting on a sorghum leaf. C‐strain and R‐strain individuals are morphologically identical. Photo by: Cesar Valencia

In our temporal collection experiments, a total of 156 moths were collected across the College Station and Lubbock locations and genotyped as per Tessnow et al. (2021) to determine strain identity. These collections included 76 moths from the early time period (0–5 h after sunset), 20 from the middle time period (5–7 h after sunset), and 60 from the late time period (7+ h after sunset). There was a significant effect of time on the probability of collecting C‐ or R‐ strain moths independent of sampling location (Lubbock χ^2^ = 18.433, df = 2, *p* < .001; College Station χ^2^ = 31.926, df = 2, *p* < .001; Combined locations χ^2^ = 102.513, df = 2, *p* < .001). C‐strain moths were collected most often early in the night (0–5 h after sunset), while the R‐strain were generally found more than 7 h after sunset, closer to dawn (Figure 4). Neither trap position nor night of collection (Lubbock only) significantly influenced the probability of collecting C‐ or R‐ strain moths (*p* > .05).

When the data were combined across both locations, we found that both factors of time (χ^2^ = 46.144, df = 2, *p* < .001) and location (χ^2^ = 20.784, df = 1, *p* < .001) had a significant effect on the probability of collecting C‐ or R‐ strain individuals. However, the interaction of time x location was not significant (χ^2^ = 0.702, df = 2, *p* = .704). This indicates that although the relative proportions of each strain differed between the two locations (College Station was 82% R‐strain and 18% C‐strain while Lubbock was 5% R‐strain and 95% C‐strain), the timing of activity between strains remains similar.

## 4 DISCUSSION

The fall armyworm is an excellent system to study how reproductive isolation may be maintained in two sympatric populations or strains. Although these strains are capable of intermating, molecular markers have not become homogenous across the strains since their identification several decades ago (Levy et al., 2002; Nagoshi, Meagher, Adamczyk, et al., 2006), suggesting they remain genetically distinct in the field despite occasional gene flow. Here, we used ddRADSeq data and temporal collection data to address two primary questions about fall armyworm strain divergence in the field: (1) To what degree do strains exhibit reproductive isolation? and (2) Do differences in daily phenology significantly contribute to reproductive isolation between strains in the field?

### 4.1 Genomic evidence of reproductive isolation

Here, we present evidence for two genetically distinct populations of fall armyworms in the central US that correlate with the previously described host‐associated strains (Pashley, 1986). Although our data clearly indicates these strains are genetically distinct, there was evidence for occasional interbreeding. Using *f3* tests, we identified seven samples that exhibited significant interstrain admixture. These samples included five putative F1 hybrids that mapped neatly in the middle of the two host strains on the PCA, and two individuals that had a mismatch between their mtDNA strain haplotype and their SNP strain genotype. This is strong evidence that hybridization occurs between the two strains in the field. Since both R‐strain and C‐strain mtDNA was recovered among the putative F1 hybrids, we conclude that in the field, fall armyworm females of both the C‐strain and the R‐strain occasionally mate with males of the opposite strain. Since two mismatch individuals also showed significant signs of admixture but appeared to be closer related to the C‐strain than the R‐strain, these individuals are predicted to be the offspring of a hybrid female of R‐strain maternal origin backcrossed to a C‐strain male.

Thirty‐two additional individuals showed a mismatch between the maternally inherited mtDNA strain marker and their SNP genotype. Although these individuals were not significantly admixed according to our *f3* test, we suspect they are the result of past hybridization events followed by several generations of backcrossing. Overtime, the signal of admixture has been reduced and can no longer be detected using *f3* statistics, however, the mismatch between the mtDNA and SNP genotype is still evident. Interestingly, 100% of moths that have a mismatch between their maternally inherited mtDNA and their SNP genotype have the R‐strain mtDNA markers. Previous studies that defined hybrids as individuals with a mismatch between the mtDNA and nuclear strain markers also found the majority of putative hybrids collected in the United States have R‐strain maternal origin (Nagoshi, 2010; Nagoshi et al., 2017; Nagoshi & Meagher, 2003; Prowell et al., 2004). This indicates that in the field hybrid females with R‐strain maternal origin are successfully mating with C‐strain males. However, hybrids with C‐strain maternal origin are not backcrossing to the R‐strain. This is strong evidence that a unidirectional barrier to reproduction exists, limiting gene flow between these strains in the field.

Although this pattern of unidirectional introgression has been routinely recovered in field data, laboratory assays have been less consistent. Some studies have found a unidirectional mating bias where R‐strain females are able to mate and produce offspring with C‐strain males, but the reverse is not true (Pashley & Martin, 1987), while other laboratory assays successfully conducted reciprocal crosses of both strains (Quisenberry, 1991; Whitford et al., 1988) and have even found that hybrid females of C‐strain maternal origin are more fertile than hybrids of R‐strain maternal origin (Kost et al., 2016). Still another study successfully conducted reciprocal interstrain crosses, but found that F1 hybrids of C‐strain maternal origin had a drastic reduction in fitness, while F1 hybrids with R‐strain maternal origin had only a minor fitness cost (Dumas et al., 2015). This last study by Dumas et al. (2015) is most consistent with our field data. Although it is not clear why variable results have been found in laboratory mating assays, these studies have consistently reported behavioral and/or physiological barriers that limit hybridization between strains, serve as barriers to gene flow, and can reinforce strain identity.

Although we detected hybridization between strains, mitochondrial markers have been used as reliable strain indicators across multiple regions of the Western Hemisphere for the past several decades (Juárez et al., 2014; Levy et al., 2002; Nagoshi, Meagher, Adamczyk, et al., 2006; Nagoshi et al., 2007). If gene flow occurs between strains and causes a mismatch between the mtDNA and SNP genotype, then these markers would be expected to homogenize across strains and become less reliable overtime. Since this has not been observed, we hypothesize that the combination of selection against unfit hybrids, a one‐way migration that removes admixture occurring north of the overwintering site, and genetic drift caused by large population size fluctuations at the overwintering site (Nagoshi & Meagher, 2004b), play a role in maintaining the genetic integrity of these strains despite occasional interstrain gene flow.

### 4.2 Temporal divergence between strains

Despite collecting fall armyworm moths in fields dominated by corn strain host plants, our initial trap captures from across the central US generally comprised both R‐strain and C‐strain individuals. This coexistence at the same location and time is consistent with previously reported trap captures across the United States (Meagher & Nagoshi, 2004; Nagoshi & Meagher, 2004a), and indicates that male moths of both the C‐ and R‐ strain routinely follow female pheromone trails to search for mates in the same fields. Although fall armyworm strains have been previously described as host plant‐associated, both strains have exhibited equal preference and performance when fed on a variety of crop types (Groot et al., 2010; Meagher et al., 2004; Pashley, 1988b; Pashley et al., 1995). This frequent overlap in habitat and mating locations combined with a lack of evidence to support strong host‐plant associations, suggests that differences in host plant use are unlikely to be the primary factor maintaining genetic differentiation between these strains.

Another factor that has been implicated in the genetic divergence of these strains is allochronic differences in mating time (Hänniger et al., 2017; Pashley et al., 1992; Schöfl et al., 2009). After observing the mating behavior of C‐ and R‐ strain fall armyworm colonies in the lab, Pashley et al. (1992) found that they exhibited strong differences in the timing of their mating activities, with the C‐ strain becoming active early in the scotophase (i.e., night) and the R‐strain becoming active much later in the scotophase (Pashley et al., 1992). This temporal difference has been observed in subsequent laboratory mating assays (Hänniger et al., 2017; Schöfl et al., 2009), and linked to heritable polymorphisms in the circadian rhythm modulator gene, *vrille* (Hänniger et al., 2017). However, this difference has never been investigated in the field. Therefore, we used temporal field collections to experimentally test the hypothesis that C‐ and R‐ strain fall armyworm moths in the field exhibit significant temporal differences in their activity periods.

We found strong evidence across both College Station, TX and Lubbock, TX that strains exhibit differences in the timing of their nightly mating activities in the field. C‐strain moths were collected in pheromone traps early in the night (0–5 h after sunset), while the R‐strain was only active later in the night more than 7 h after sunset (Hänniger et al., 2017; Pashley et al., 1992; Schöfl et al., 2009). Although C‐ and R‐ strain moths were both collected at each of our sampling locations, the population composition differed. In College Station, TX, the majority of the population were of the R‐strain while in Lubbock, TX, most of the population was made up of the C‐strain. Despite these differences, there was no interaction between location and time. This is strong evidence that regardless of the number of C‐ or R‐strain individuals present in a population, the temporal differences in activity between strains persist. This clear shift in daily phenology between strains in wild fall armyworm populations confirms previous lab observations and provides a critical pre‐zygotic isolating mechanism that likely reduces interstrain hybridization while these moths are residing in the same fields.

Although all moths collected in the pheromone traps in this study were males, laboratory studies have indicated that male nightly activity periods are more labile than those of females (Pashley et al., 1992). Thus, it is likely that females are exhibiting similar if not stronger differences in activity patterns in the field. Our data strongly support allochrony as a critical prezygotic reproductive isolating mechanism underlying fall armyworm strain differentiation in the field. In light of our data, there are now several lines of evidence to suggest that referring to fall armyworm strains as ‘host‐associated’ may be ecologically inaccurate. First, behavioral studies have failed to consistently find differences in host plant preference and performance between the strains (Groot et al., 2010; Juárez et al., 2014; Meagher et al., 2004; Pashley, 1988b; Pashley et al., 1995). Second, clear phenological differences have been routinely observed in the lab (Hänniger et al., 2017; Pashley et al., 1992; Schöfl et al., 2009). And lastly, differences in nightly activity patters between the two strains have now been confirmed in the field. Thus, the combined evidence indicates that it may be more accurate to refer to these two genetically distinct fall armyworm populations as allochronic strains.

## CONFLICT OF INTEREST

The authors declare no conflicts of interest.

## AUTHOR CONTRIBUTIONS

**Ashley E. Tessnow:** Conceptualization (lead); data curation (equal); formal analysis (equal); funding acquisition (equal); investigation (equal); methodology (equal); project administration (equal); writing – original draft (lead); writing – review and editing (equal). **Tyler J.Raszick:** Conceptualization (supporting); formal analysis (equal); methodology (supporting); writing – review and editing (equal). **Patrick Porter:** Investigation (equal); methodology (supporting); resources (supporting); writing – review and editing (equal). **Gregory A. Sword:** Conceptualization (equal); data curation (supporting); formal analysis (supporting); funding acquisition (lead); investigation (equal); methodology (equal); project administration (supporting); resources (lead); supervision (lead); validation (equal); writing – review and editing (equal).

## Data Availability

All raw sequence reads have been made available through the NCBI sequence read archive (SRA). These sequences can be retrieved using the bioproject accession numbers PRJNA645462 or can be accessed at the following link https://www.ncbi.nlm.nih.gov/bioproject/?term=PRJNA645462. Additionally, all information about our SNP dataset and the moths collected to assess temporal differences in strain activity has been made available in the Dryad Digital repository and can be found using the following DOI accession number: 10.5061/dryad.4qrfj6qc1.

## 1

**TABLE A1:** List of the most abundant host plants surrounding each trap in the field collection experiment. There was no effect of trap on the proportion of C‐ and R‐ strain moths collected

	Trap location and ID															
Plant	Lubbock, TX						College Station, TX									
	LB‐1	LB‐2	LB‐3	LB‐4	LB‐5	LB‐6	CS‐1	CS‐2	CS‐3	CS‐4	CS‐5	CS‐6	CS‐7	CS‐8	CS‐9	CS‐10
Corn							X	X	X		X			X	X	
Cotton		X	X	X	X	X		X		X			X	X		
Peanut		X	X		X											
Sorghum (Booting)						X	X	X	X	X		X			X	X
Sorghum (Mature)	X		X	X												
Soybean		X														
Sunflower	X															
Weeds							X			X	X		X			
# Moths collected	1	37	9	17	4	16	0	3	0	4	0	8	3	17	14	6

**TABLE A2:** Chromosome position and NCBI BLAST matches for sequences containing SNPs that appeared as outliers (high *F*_st_ values) on the Manhattan plot (Figure 3a). Many of the SNPs that showed high levels of divergence were clustered into six groups. The remaining are listed as singletons in the bottom of the table

SNP ID	Group	Chrom	Position (bp)	*F*_st_	NCBI BLAST match	Mutation notes^a^	NCBI Reference
195	1	1/Z	10,927,561	0.78	No significant match found	N/A	N/A
197			10,927,579	0.78			
201	2	1/Z	11,030,881	0.77	Homologous to a protein coding region for a suppressor of cytokine signaling (SOCS) protein in *S*. *litura*. GO molecular function: 1‐phosphatidylinositol‐3‐kinase regulator activity	Synonymous	XM_022977237.1, XM_022977236.1, XM_021332332.1, XM_021332331.1
203			11,030,973	0.77		Non‐synonymous TYR to HIS	
205			11,030,998	0.77		Synonomous	
207			11,031,097	0.77		CYS to stop codon	
208			11,031,103	0.77		Synonomous	
209			11,031,124	0.77		Synonomous	
210			11,031,163	0.78		Synonomous	
211			11,031,172	0.78		Synonomous	
222	3	1/Z	12,671,645	0.72	No significant match found	N/A	N/A
223			12,671,651	0.73			
227			12,671,837	0.73			
250	4	1/Z	13,292,971	0.77	Protein coding region of predicted Spodoptera litura 1‐phosphatidylinositol 4,5‐biphosphate phosphodiesterase gamma‐1	Synonomous	XM_022976465.1, XM_022976466.1
253			13,293,046	0.76		Synonomous	
254			13,293,061	0.77		Synonomous	
258			13,295,934	0.74		Synonomous	
264			13,296,117	0.77		Synonomous	
418		1/Z	22,985,972	0.76	No significant match found	N/A	N/A
422			22,986,192	0.76			
423	5		22,986,226	0.76			
424			22,986,239	0.76			
425			22,986,294	0.76			
16	Singletons	1/Z	1,121,015	0.73	Predicted coding region for kalirin protein in *S*. *litura*	Synonymous	XM_022976921.1
106		1/Z	4,933,322	0.78	Matched to *S*. *litura* uncharacterized mRNA	N/A	XM_022960480.1
301		1/Z	14,104,488	0.79	No significant match found	N/A	N/A
307		1/Z	14,412,021	0.72	Upstream of coding region for LIM/homeobox protein Lhx2‐like	N/A	XM_026891428.1, XM_021330118.1, XM_022977019.1
2757		12	12,456,628	0.72	No significant match found	N/A	N/A
3686		16	14,134,047	0.74	Protein coding region of Solute carrier family 25 member 35‐like isoform in *S*. *litura*	Synonomous	XM_022963959.1

Note

^a^ If SNP Fst values were >0.7 and matched to a coding region in the genome, mutation notes indicate if the SNP is predicted to result in a synonymous or nonsynonmous mutation.
